# Comparative Effectiveness of Collaborative Treatment with Korean and Western Medicine for Low Back Pain: A Prospective Cohort Study

**DOI:** 10.1155/2021/5535857

**Published:** 2021-07-28

**Authors:** Hye-Yoon Lee, Min Kyoung Cho, NamKwen Kim, Se Yeon Lee, Na-Gyeong Gong, Eun Hye Hyun

**Affiliations:** ^1^Research Institute for Korean Medicine, Pusan National University, Yangsan 50612, Republic of Korea; ^2^Department of Medical Education, School of Medicine, Pusan National University, Yangsan 50612, Republic of Korea; ^3^School of Korean Medicine, Pusan National University, Yangsan 50612, Republic of Korea

## Abstract

In Korea, low back pain is the ailment that is most frequently treated using collaborative care regimens that include aspects of Western and traditional Korean medicine. As part of a national pilot project on the collaboration between Western and Korean medicine, we aimed to investigate the clinical effectiveness of collaborative treatment and compare it with treatment methods that involved only Korean or Western Medicine practices for patients with low back pain. This nationwide, multicenter, prospective, observational, and comparative study spanned 8 weeks, during which patients with low back pain were evaluated at three time points (at baseline, 4 weeks, and 8 weeks). The primary outcome was low back pain-related disability measured by the Oswestry Disability Index, while the secondary outcomes included severity of low back pain (as on a numeric rating scale) and quality of life (as per a 5-level EuroQol-5 dimensions questionnaire). We analyzed 150 patients (including 129 per-protocol cases) and found that the Oswestry Disability Index and 5-level EuroQol-5 dimensions showed statistically significant differences over time between the collaborative treatment group and the sole treatment group after adjusting for sex, income level, and age. Conversely, the numeric rating and EuroQol-visual analog scales showed no significant between-group differences over time. Based on our findings, we believe that collaborative treatment that includes parallelly administered aspects of Western and Korean medicine can benefit patients with low back pain by facilitating functional improvements and lead to a better quality of life.

## 1. Introduction

Low back pain (LBP) affects 540 million people across the world [1] and the loss of working hours due to LBP increased by 54% in 2015 compared to 1990 [2]. A multidisciplinary approach is imperative for treating LBP effectively [3], which should ideally include a combination of pharmacological and nonpharmacological treatments that result in functional recovery of muscles and tendons [4, 5].

In South Korea, LBP is one of the diseases most frequently treated using traditional Korean Medicine (KM), which is often administered parallelly with Western Medicine (WM) or as a gradual successive step after a WM regimen [6]. Korea has a dual medical system wherein WM doctors (MDs) and KM doctors (KMDs) operate cohesively. This dual system is advantageous because it increases patient satisfaction and expands the range of treatment options [7, 8]; however, its pitfalls are that the medical costs increase due to redundant treatments from both systems and the treatment modality becomes dependent on the patients' choices, which increases the conflict between the KM and WM systems [9, 10].

To address these concerns, the Korean Ministry of Health and Welfare launched the “WM-KM collaborative treatment (CT) pilot project” to investigate the utility of collaborative treatment (CT), develop an ideal CT model for each disease, and evaluate the clinical efficacy and cost-effectiveness of CT. Initiated in November 2017, this second-stage project aims to assess the feasibility of providing a “collaboration fee” to the participating institutions from the National Health Insurance System as an additional reimbursement. This fee seeks to facilitate collaboration between MDs and KMDs by applying insurance coverage to both WM and KM treatments when a patient is treated by both an MD and KMD for the same disease on the same day.

In conjunction with this pilot project, we aimed to conduct a prospective observational analysis of the Registry for Korean Medicine and Western Medicine Collaborative Treatment (REKOMENT) to compare the clinical effectiveness of CT to that of sole treatment (ST) with WM or KM for patients with LBP––an ailment that is largely treated using CT in Korea.

## 2. Methods

### 2.1. Study Design and Setting

This multicenter, prospective, observational study targeted all LBP patients who visited any of the hospitals participating in the WM-KM collaborative treatment pilot project from 7 November 2017 to 14 October 2019. Four university-affiliated hospitals and three KM hospitals participated in this research project and each institution's IRB approved the study: Korean Medicine Hospitals of Dongguk University (DUBOH-IRB 2018-0002), Daejeon University (DJDSKH-18-BM-06), Dongshin University (DSMPOS18-2), and Gachon University (18-104) and Design Hospital (P01-201806-21-001), Samse Hospital (P01-201807-21-004), and the Mokhuri Hospital (P01-201807-21-011). The study design was registered at “Clinical Research Information Service” (CRIS) as a prospective study, and the details can be found in the published study protocol [11]. The entire research process was conducted in accordance with the Declaration of Helsinki and the Good Research Practices recommended by the International Society for Pharmacoeconomics and Outcomes Research (ISPOR).

### 2.2. Participants

This study included LBP patients over the age of 19 who visited any of the aforementioned clinics for the first time and voluntarily provided their written informed consent to participate. The patients who were currently participating in another clinical trial, or found it difficult to comply with the study schedule, or comprehend and respond to study questionnaires were excluded.

Announcements regarding the study were displayed publicly at the institutions to facilitate equal-opportunity participation.

### 2.3. Patient Groups

As this was an observational study, participants were treated using individualized treatment plans that were designed according to their specific disease condition, the duration and severity of LBP, and underlying diseases, if any. The Korean Medicine clinical practice guideline outlines the general treatment principles for chronic LBP [12], and in our study, the attending MD or KM used their expertise to decide whether a particular participant should receive CT or ST (with either WM or KM). More details regarding the treatment methods are outlined in “clinical pathways for collaborative treatment of low back pain” provided as a supplementary file. ST treatment methods are also included in the CT pathways. Participants who received CT were assigned to the CT group and those who received ST were assigned to the ST group. We assessed differences between the CT and ST groups by comparing their baseline characteristics.

The participants received routine care and were neither subjected to nor denied any specific treatments by partaking in this study. Afterwards, those patients who had incurred a collaboration fee even once over the course of the study were categorized and analyzed as part of the CT group. A collaboration fee required a written proof of collaborative care provided by both the MD and the KMD who treated the patient for the same disease on the same day.

### 2.4. Study Blinding

The subjects and practitioners (KMs and MDs) could not be blinded because of the observational design of the study. However, the evaluators who conducted the assessment and performed the statistical analysis were blinded.

### 2.5. Outcome Measurements

The participants undertook surveys that were conducted at three time points: immediately after initial treatment (baseline), after 4 weeks, and after 8 weeks. The baseline characteristics were collected during the first survey and clinical indicators were evaluated in every survey. The baseline surveys were conducted by researchers from each institution via face-to-face interviews, while the second and third surveys were conducted by one researcher via a phone interview. All the surveys were conducted using a well-defined questionnaire and lasted 5–10 minutes each.

### 2.6. Primary Outcome

The primary endpoint in this study was the Oswestry Disability Index (ODI) score, which captures the intensity of LBP and the degree of LBP-related disability caused in daily life [13]. The ODI measures 10 parameters, namely, pain intensity, personal care, lifting, walking, sitting, standing, sleeping, sex life, social life, and traveling [14]. The Minimal Important Difference (MID) refers to the minimum clinically important difference that measures changes in the patient's condition [15]; for the ODI, the MID was set at 10 points [16]. In this study, we used the Korean version of the ODI proposed by Kim et al. [17].

### 2.7. Secondary Outcomes

The secondary endpoints in this study were the numeric rating scale (NRS), 5-level EuroQol-5 dimensions (EQ-5D-5L), and EuroQol-visual analog scale (EQ-VAS) measurements.

NRS is an index that converts the LBP-related pain intensity into numerical values using the patient's verbal rating of their LBP. It is scored between 0 (no pain) and 10 (worst pain imaginable) [18]. The MID of NRS for LBP was established at 2 points [16, 19].

EQ-5D-5L measures the patient's health-related quality of life (HRQOL) [20], which is rated according to five response levels to questions in five dimensions, namely, mobility, self-care, usual activity, pain/discomfort, and anxiety/depression. We used the officially verified Korean version of the EQ-5D-5L [21].

EQ-VAS calculates the overall health level of the patient using a vertical scale that ranges from 0 to 100 points [22]. We calculated the utility value of HRQOL and analyzed it using the recently reported national tariff, which was developed and published to translate the five domain values of EQ-5D into utility values that could be scored between 0 (dead) and 1 (perfect health) [23].

The higher the value calculated using EQ-5D-5L, the higher was the HRQOL. The MID of EQ-5D and EQ-VAS for chronic LBP were set at 0.08 and 10.5, respectively [19, 24].

### 2.8. Statistical Analysis

Continuous variables were analyzed after being tested for normality using the Shapiro–Wilk test. Differences in categorical variables between groups were analyzed using the Chi-square and Fisher's exact tests. Between-group differences in the means of continuous variables were analyzed using the Student's *t*-test and the Mann–Whitney *U* test. Differences in more than three groups were analyzed using analysis of variance (ANOVA) and the Kruskal–Wallis test.

Observations for the primary endpoint were presented as per-protocol (PP) data, and the analysis of the difference in mean variations between the two groups during the observation period was presented by performing repeated-measures ANOVA. The clinical effectiveness was analyzed using intention-to-treat (ITT) data. The amount and mechanisms of missing data were verified after processing the missing items using multiple imputation (MI). The final results were generated following analysis with the Generalized Linear Mixed Effect Model (GLMM), a random-effects model used to analyze longitudinal data.

All data analyses were performed using the Stata MP version 14.2 software (StataCorp, Texas, USA). The statistical significance was considered at a *P* value of <0.05.

## 3. Results

### 3.1. Participants

**Figure 1:**
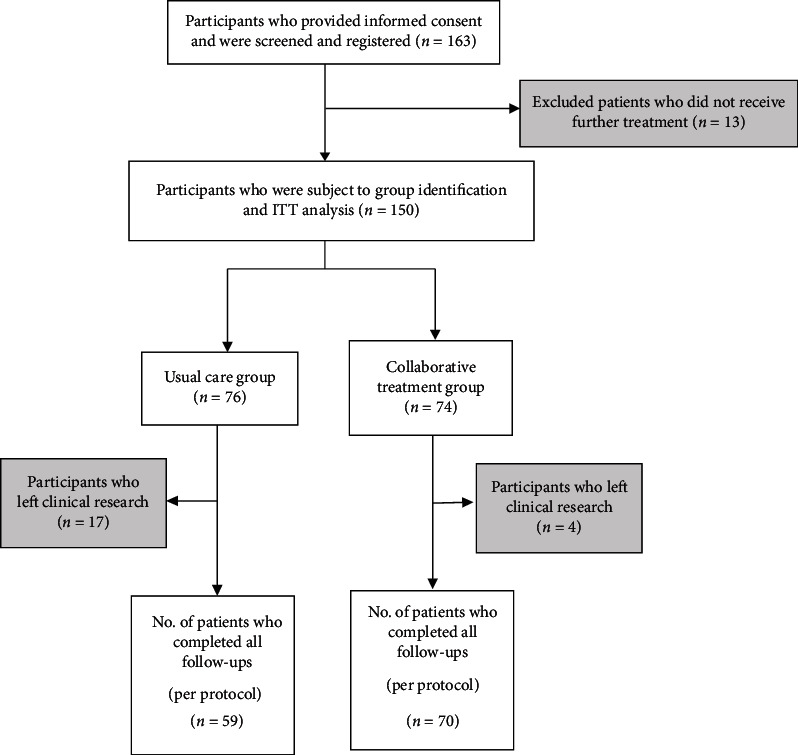
Flow diagram of patient selection.

We screened and registered 163 patients, of which 13 were excluded because they visited the hospital only once. Then, we analyzed 150 patients as ITT subjects, but only 129 had complete data with no missing items. These 129 patients became the PP subjects for our clinical effectiveness analysis. Of the 150 patients included in the ITT analysis, 74 patients had received both WM and KM treatments so they were labelled as the CT group, and the rest of the 76 patients who received only usual care were labelled as the ST group. Four patients in the CT group and 17 patients in the ST group were excluded from the PP analysis because of missing data (Figure 1). Of the 21 patients with missing data, 1 was missing an evaluation variable in the baseline survey, 18 were unable to complete the second and third surveys, and 2 were lacking in their hospital medical records and administrative data.

### 3.2. Baseline Characteristics of Patients

There were no significant differences between the two groups according to age, diagnosis, NRS, and EQ-VAS values; however, the baseline ITT analysis showed significant between-group differences according to sex, income level, and ODI and EQ-5D-5L measurements. We used these baseline differences to develop an imputation model and analyze the effectiveness of the GLMM (Table 1).

### 3.3. Missing Patient Data

The final proportion of patients with missing data in terms of the main variables of primary and secondary endpoints was 14.7%, with the missing data representing 8.0% of the total data. We performed Little's test to check missing mechanisms. The results showed a missing completely at random (MCAR) mechanism (Chi-square value = 48.2677, *P* value = 0.3813). On verifying the covariate association of the base variables (sex, income level, and age), we observed an estimated covariance-dependent missing completely at random (CD-MCAR) mechanism. Thus, after processing the missing data by MI, which sets the base variables as explanatory variables, we used ITT data for our analysis (Table 2).

### 3.4. Effectiveness Outcomes

#### 3.4.1. Mean Changes in Outcomes during the Study Period (PP)

Primary outcome: the measured ODI was 27.01 ± 15.28 in the ST group and 35.49 ± 14.98 in the CT group immediately after initial treatment. After 4 weeks, the ODI was 22.37 ± 15.30 in the ST group and 21.29 ± 12.37 in the CT group. After 8 weeks, the ODI was 19.81 ± 15.08 in the ST group and 17.52 ± 15.68 in the CT group.

**Figure 2:**
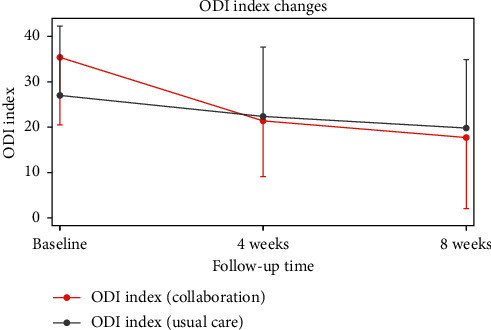
Mean changes in ODI during the follow-up period. ODI: Oswestry Disability Index.

These results indicated that both groups experienced a statistically significant reduction in ODI over time (within-group effects). This reduction also showed statistically significant between-group differences, which suggested that the degree of disability caused by LBP reduced markedly in the CT group compared to the ST group (*P* < 0.001) (Table 3, Figure 2).

Secondary outcomes: the measured NRS was 5.15 ± 1.89 in the ST group and 5.41 ± 1.82 in the CT group immediately after initial treatment. After 4 weeks, the NRS was 3.29 ± 1.88 in the ST group and 2.93 ± 1.63 in the CT group. After 8 weeks, the NRS was 2.64 ± 1.99 in the ST group and 2.51 ± 2.10 in the CT group. These results showed that both groups experienced a statistically significant reduction in NRS over time (within-group effects). However, there were no significant between-group differences over time.

The measured EQ-5D-5L was 0.74 ± 0.13 in the ST group and 0.68 ± 0.15 in the CT group immediately after initial treatment. After 4 weeks, EQ-5D-5L was 0.83 ± 0.11 in the ST group and 0.84 ± 0.10 in the CT group. After 8 weeks, EQ-5D-5L was 0.87 ± 0.14 in the ST group and 0.89 ± 0.12 in the CT group. The results indicate that both groups experienced a statistically significant increase in EQ-5D-5L over time, both within each group and between the two groups. This suggests that the HRQOL increased markedly in the CT group compared to the ST group (between-group effects) (*P*=0.001).

**Figure 3:**
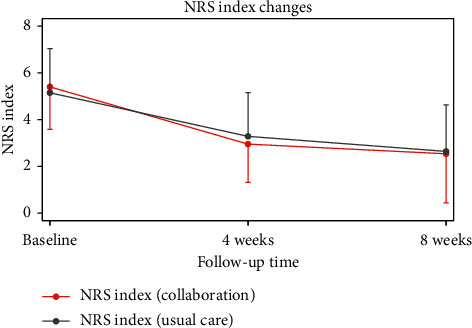
Mean changes in NRS during the follow-up period. NRS: numeric rating scale.

**Figure 4:**
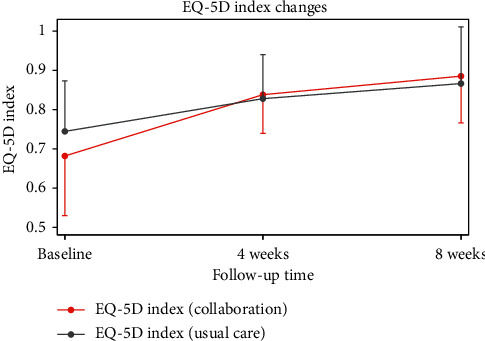
Mean changes in EQ-5D-5L during the follow-up period. EQ-5D-5L: 5-level EuroQol-5 dimensions.

**Figure 5:**
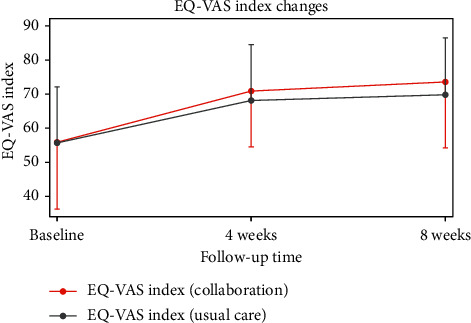
Mean changes in EQ-VAS during the follow-up period. EQ-VAS: EuroQol-visual analog scale.

The measured EQ-VAS was 55.69 ± 16.4 in the ST group and 55.84 ± 19.79 in the CT group immediately after initial treatment. After 4 weeks, the EQ-VAS score was 68.14 ± 16.40 in the ST group and 71.20 ± 16.32 in the CT group. After 8 weeks, the EQ-VAS score was 69.81 ± 16.72 in the ST group and 73.91 ± 19.27 in the CT group. The results showed that both groups experienced a statistically significant increase in EQ-VAS over time (within-group effects), but there were no significant between-group differences (Table 3, Figures 3–5).

#### 3.4.2. Effectiveness Outcomes When Adjusting Covariates (ITT)

After verifying and performing MI on the missing data, we observed that the clinical effectiveness of both types of treatments had changed over time. We analyzed the between-group differences in this change using GLMM. When we adjusted the variables of sex, income level, age, and diagnosis group, ODI and EQ-5D-5L showed statistically significant differences between the two groups over time. Conversely, NRS and EQ-VAS scores showed no between-group differences over time (Table 4).

## 4. Discussion

A recent study used national health insurance data in Korea to analyze the medical costs of WM and KM for patients with joint diseases and revealed that the largest portion of WM costs were attributed to physiotherapy and more than 70% of KM costs referred to medical procedures, such as acupuncture, moxibustion, and cupping [25]. Even though WM and KM apply different treatments for the same disease, the study found that patients who received both WM and KM treatments for the same disease on the same day could not receive insurance coverage for these procedures, which burdened them with medical expenses. However, this should not be the norm because medical institutions in Korea have been legally allowed to perform CT since 2010 and the use of CT has been growing [26].

In this study, we defined CT as a medical treatment that combines aspects of WM and KM and is administered in a hospital setting by both WM and KM doctors who share a patient's medical information and consult each other to plan the treatment [27]. For example, if an MD decides that a patient requires KM treatment in addition to analgesic medicine and physical therapy, the MD can consult a KMD and collaboratively design a treatment plan that includes some KM techniques to enhance the effectiveness of the overall treatment and avoid duplication.

The first-stage trial project of REKOMENT that started in July 2016 revealed that, currently, LBP is the ailment that is most frequently treated using CT in Korea [7]. In this study, we enrolled a wide pool of patients who are participating in the ongoing “WM-KM CT pilot project” in Korea and analyzed patient data from REKOMENT to assess the clinical effectiveness of CT on LBP. Therefore, the participants' disease duration, severity, cause, and accompanying symptoms varied greatly, and we set the observation period to 8 weeks to evaluate the short-term effectiveness of CT in its early phase of administration.

We also examined the factors that influenced each patient's decision to undergo CT. The participants' baseline characteristics showed that those with a relatively severe LBP-related disability and a low quality of life tended to choose CT. This finding is consistent with those of previous studies, which showed that patients with spinal cord disease (who usually experience severe LBP) tended to opt for KM treatments, such as acupuncture, moxibustion, cupping, and Chuna therapy [28]. Further, patients with limiting LBP chose acupuncture and chiropractic treatments more often than those with nonlimiting LBP [29]. However, since our study was conducted in seven CT-focused institutions, it was difficult for us to accurately convey the general situation of CT use in Korea. Therefore, comprehensive, large-scale studies that include standard medical institutions should be conducted to understand the general utility of CT and the main factors that influence patient decisions.

In terms of clinical effectiveness, our study showed that patients in both the CT and ST groups experienced significant positive changes in pain intensity, daily-life disability, and HRQOL. However, we observed that CT was more effective than ST in reducing disability and improving the HRQOL.

A clinical study on chronic back pain stated that it was clinically significant if the NRS score decreased by more than 2.4 points [30] or varied by more than 20% between two time points [31, 32]. In our study, LBP significantly reduced in intensity in both the CT and ST groups. When patients self-assess chronic pain, they tend to be influenced by nonpainful factors, such as experience and emotions regarding the disease condition and treatment, and they consider pain intensity as a comparative rather than a linear index [33]. These factors were likely to have played a role in our study because it was not a double-blind study. Nonetheless, ODI, which measures the degree of disability caused by LBP, evaluates pain intensity according to a five-point scale using questions on daily life, physical activity, and social life. [34]. The ODI questions are more detailed than those of NRS and the answer choices are relatively objective (e.g., walking distance, standing time, and sleeping time); therefore, it is likely that the patients' conditions are reflected objectively and in more detail. Furthermore, patients with musculoskeletal diseases tend to restrict specific movements to avoid pain; therefore, while evaluating pain relief, it is important to consider not only pain intensity but also limitations in the patient's overall function [35].

Chronic LBP is caused by neuroplastic changes in sensorimotor control; thus, cognitive-based interventions, such as education and physical interventions, have the potential for clinical use [36]. Acupuncture, a major treatment in KM, induces peripheral sensory stimulation and is considered a bottom-up physical intervention to address neuroplastic changes. Therefore, for patients with chronic LBP, acupuncture might improve impaired somatosensory processing by improving tactile precision and reduce grey matter to facilitate greater improvement and increased fractional anisotropy in the posterior cortical somatosensory region [37]. Although there was no significant difference between the two groups in our study, findings from the cited studies warrant further research on chronic pain.

There are several limitations to this study. First, this was not a randomized controlled study and the patients were not blinded. So, we could not rule out all the factors that may influence the results, including placebo effects. Therefore, the findings of this study cannot be used to draw definite conclusions on the efficacy of ST or CT. Second, this research included all patients whose chief complaint was LBP; thus the diseases that caused the LBP may vary greatly. We used statistical analysis methods to exclude the effects of age, the severity of symptoms, and diagnosis, in order to reveal the impact of ST and CT. Since this was a multicenter study, the differences between each clinician's technique and treatment method contributed to the heterogeneity of the results. However, this study analyzed the hospitals following CT protocols as part of the “WM-KM CT pilot project.” Therefore, we consider the variation in treatment methods and techniques to be minimal and unsubstantial. However, further studies focusing on a more specific causative disease are needed in the future. Third, as a multicenter, prospective cohort study, the number of patients we included was relatively small, which makes it difficult to generalize our results. To address these limitations, large-scale controlled trials are needed in the future to investigate the clinical effectiveness of CT on LBP in more detail.

Overall, in our study, patients who were treated for LBP using CT showed significant improvement in daily-life disability and HRQOL compared to those who received ST, which means that CT provides additional benefits to LBP patients and aids in faster recovery.

## Figures and Tables

**Table 1:** Baseline characteristics of patients.

Variables		Usual care *N* = 76		Collaboration *N* = 74		*P* value
Sex	Male	24	31.58%	39	52.7%	0.009^*∗∗*^
	Female	52	68.42%	35	47.3%	
Income (monthly) (won)	Unknown	54	71.05%	41	55.41%	0.003^*∗∗*^
	Under 2 million	5	6.58%	4	5.41%	
	2–5 million	8	10.53%	26	35.14%	
	5–10 million	7	9.21%	2	2.7%	
	Over 10 million	2	2.63%	1	1.35%	
Age (years old)		44.39	17.33	48.78	13.9	0.0896
Diagnosis	Dorsalgia	37	48.68%	33	44.59%	0.825
	Sprain	29	38.16%	29	39.19%	
	HIVD and others	10	13.16%	12	14.67%	
ODI		26.75	15.32	35.44	14.98	0.0006^*∗∗*^
NRS		5.066	2.016	5.466	1.80	0.2036
EQ-5D-5L		0.7344	0.1444	0.6786	0.1522	0.0226^*∗∗*^
EQ-VAS (mm)		54.46	16.65	55.03	20.23	0.8527
Duration (day)		198.8	820.90	85.53	465.40	0.3019

^*∗*^Frequency, mean, and percent or standard deviation; ^*∗∗*^*P* value < 0.05; HIVD: herniated intervertebral disc; ODI : Oswestry Disability Index; NRS: numeric rating scale; EQ-5D-5L: 5-level EuroQol-5 dimensions; EQ-VAS : EuroQol-visual analog scale.

**Table 2:** Summary of censored data for outcome variables.

Variables	Never censored	Ever censored	Total
Participant (N)			
Control group	59	17	76
Case group	69	5	74
Total	128	22	150
Participant (%)			
Control group	77.63	22.37	100.00
Case group	93.24	6.76	100.00
Total	85.33	14.67	100.00
Potential periods of observation (day)			
Control group	708	204	912
Case group	828	60	888
Total	1536	264	1800
Periods with data available (day)			
Control group	708	85	793
Case group	828	35	863
Total	1536	120	1656
Periods with data available (%)			
Control group	100.00	41.67	86.95
Case group	100.00	58.33	97.18
Total	100.00	45.45	92.0
Total percent of the periods with censored data (%)	0	54.55	8.00
Total percent of patient numbers censored			14.67%
		Chi-square distance	*P* value
MCAR test result		48.26	0.38
MCAR (CDM) test result		102.77	1.00

MCAR: missing completely at random; CDM: covariate dependent missingness.

**Table 3:** Mean changes in the outcomes of effectiveness per group over time and the results of repeated-measures Analysis of Variance (per-protocol).

Outcome measure	Treatment group	Baseline		4 weeks		8 weeks		RM ANOVA		
		Mean	SD	Mean	SD	Mean	SD	Group	Time	Group × time
ODI	Usual care	27.01	15.28	22.37	15.30	19.81	15.08	0.438	<0.001^*∗*^	<0.001^*∗*^
	Collaboration	35.49	14.98	21.29	12.37	17.52	15.68			
NRS	Usual care	5.15	1.89	3.29	1.88	2.64	1.99	0.763	<0.001^*∗*^	0.200
	Collaboration	5.41	1.82	2.93	1.63	2.51	2.10			
EQ-5D-5L	Usual care	0.74	0.13	0.83	0.11	0.87	0.14	0.555	<0.001^*∗*^	0.001^*∗*^
	Collaboration	0.68	0.15	0.84	0.10	0.89	0.12			
EQ-VAS	Usual care	55.69	16.45	68.14	16.40	69.81	16.72	0.302	<0.001^*∗*^	0.482
	Collaboration	55.84	19.79	71.20	16.32	73.91	19.27			

^*∗*^ *P* value < 0.05; SD: standard deviation; ODI: Oswestry Disability Index; NRS: numeric rating scale; EQ-5D-5L: 5-level EuroQol-5 dimensions; EQ-VAS: EuroQol-visual analog scale. For ODI and NRS, the decreased amount indicates the degree of improvement of the symptoms (and how effective the treatment was). For EQ-5D and EQ-VAS, the increased amount indicates the same as above.

**Table 4:** Results of the generalized linear mixed model analysis of effectiveness outcomes (intention-to-treat).

Random effects	ODI		NRS		EQ-5D-5L		EQ-VAS	
	*β*	SE	*β*	SE	*β*	SE	*β*	SE
Collaboration	11.98^*∗*^	3.37	0.36	0.44	−0.08^*∗*^	0.03	−2.06	4.27
Follow-up time	−3.54^*∗*^	1.08	−1.25^*∗*^	0.15	0.07^*∗*^	0.01	7.37^*∗*^	1.42
Collaboration × follow-up time	−5.17^*∗*^	1.49	−0.19	0.20	0.04^*∗*^	0.01^*∗∗*^	1.70	1.98
Sex	0.38	2.08	0.13	0.26	−0.02	0.02	−5.66^*∗*^	2.09
Age	0.12	0.07	0.02^*∗*^	0.01	0.00	0.00	−0.04	0.07
Diagnosis (vs. sprain)	−1.58	2.24	−0.31	0.28	−0.01	0.02	−1.12	2.24
(vs. HIVD)	−2.12	3.00	0.47	0.37	−0.02	0.02	−3.37	2.99
Intercept	24.59^*∗*^	3.89	5.08^*∗*^	0.50	0.72^*∗*^	0.03	54.62^*∗*^	4.38

^*∗*^ *P* value < 0.05; ODI : Oswestry Disability Index; NRS: numeric rating scale; EQ-5D: 5-level EuroQol-5 dimensions; EQ-VAS : EuroQol-visual analog scale; SE: standard error; HIVD: herniated intervertebral disc. Reference group of diagnosis dummy variable is dorsalgia.

## Data Availability

The data used to support the findings of this study are available from the corresponding author upon request.

## Supplementary Materials

Supplementary Materials The supplementary file includes four clinical pathways depending on the disease entity associated with low back pain: (1) clinical pathway for collaborative treatment of low back pain, (2) clinical pathway for collaborative treatment of low back sprain, (3) clinical pathway for collaborative treatment of lumbar herniated intervertebral disc, and (4) clinical pathway for collaborative treatment of lumbar intervertebral disc disorder. Such variables as symptom severity and disease entity were controlled using statistical methods.
