# Supplementary material for: Comparative Effectiveness of Collaborative Treatment with Korean and Western Medicine for Low Back Pain: A Prospective Cohort Study
Source: Evid Based Complement Alternat Med. 2021 Jul 28;2021:5535857. doi: 10.1155/2021/5535857 (PMC8342155; doi:10.1155/2021/5535857)
Supplement: Supplementary Materials — The supplementary file includes four clinical pathways depending on the disease entity associated with low back pain: (1) clinical pathway for collaborative treatment of low back pain, (2) clinical pathway for collaborative treatment of low back sprain, (3) clinical pathway for collaborative treatment of lumbar herniated intervertebral disc, and (4) clinical pathway for collaborative treatment of lumbar intervertebral disc disorder. Such variables as symptom severity and disease entity were controlled using statistical methods. [file 5535857.f1.pdf]

# Clinical Pathway for collaborative treatment between Korean medicine and Western medicine

## 1. Clinical pathway for collaborative treatment of low back pain

-Suwon Mokhuri Korean Medicine Hospital

<Clinical Pathway process>

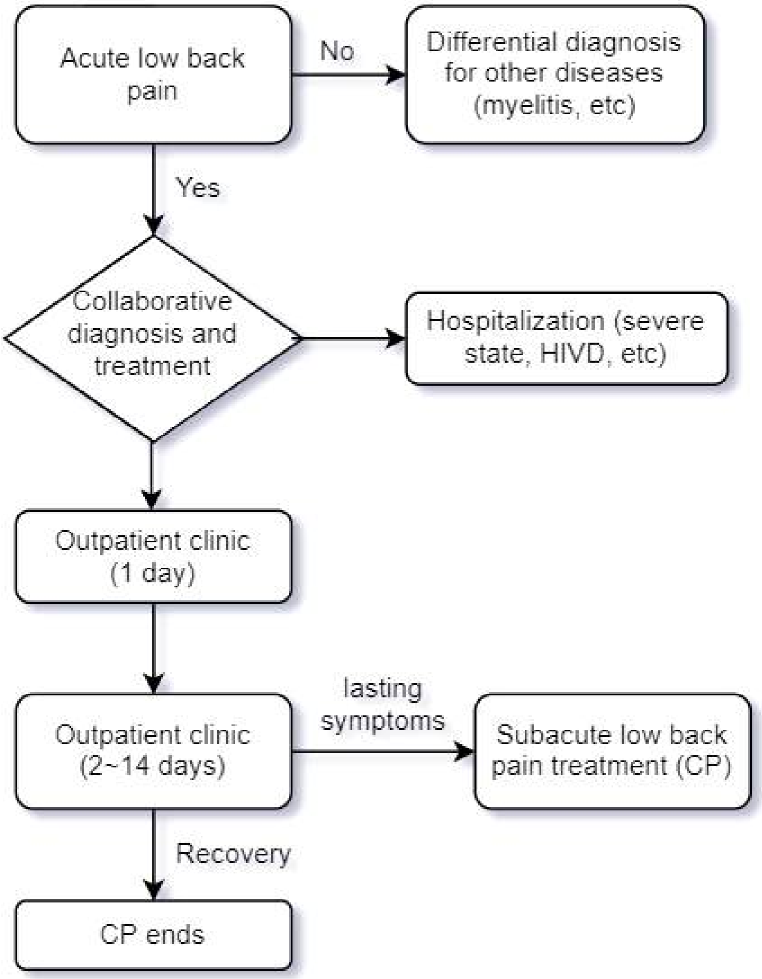

<Treatment Plan>

| | Day 1 | Day 2~7 | Day 8~14 |
|---|---|---|---|
| 1. Evaluation & observation | 1. Past medical history, treatment history<br>2. Evaluation of the severity of pain<br>3. Evaluation of daily function (ODI) | 1. Evulation of the severity of pain (VAS)<br>2. Evaluation of daily fundtion (ODI) | 1. Evulation of the severity of pain (VAS)<br>2. Evaluation of daily fundtion (ODI) |
| 2. Diet | Ordinary diet (no drinking or smoking) | Ordinary diet (no drinking or smoking) | Ordinary diet (no drinking or smoking) |
| 3. Examination | 1. X-ray<br>2. L-spine MRI | | |
| 4. Prescription | 1. WM : analgesic, antiphlogistic<br>2. KM : herbal medicine | 1. WM : analgesic, antiphlogistic<br>2. KM : herbal medicine | 1. WM : analgesic, antiphlogistic<br>2. KM : herbal medicine |
| 5. Treatment | 1. WM : physical therapy (electric stimulation), kinesitherapy<br>2. KM : acupuncture, KM physiatrics | 1. WM : physical therapy (electric stimulation), kinesitherapy<br>2. KM : acupuncture, KM physiatrics | 1. WM : physical therapy (electric stimulation), kinesitherapy<br>2. KM : acupuncture, KM physiatrics |
| 6. Education and explanation | 1. Explain treatment and prognosis<br>2. Lower back stretching education<br>3. Lower back muscle strengthening education | 1. Explain treatment and prognosis<br>2. Lower back stretching education<br>3. Lower back muscle strengthening education | 1. Explain treatment and prognosis<br>2. Lower back stretching education<br>3. Lower back muscle strengthening education |

## 2. Clinical pathway for collaborative treatment of low back sprain

- Kyung Hee University Korean Medicine Hospital at Gangdong

<Clinical Pathway process>

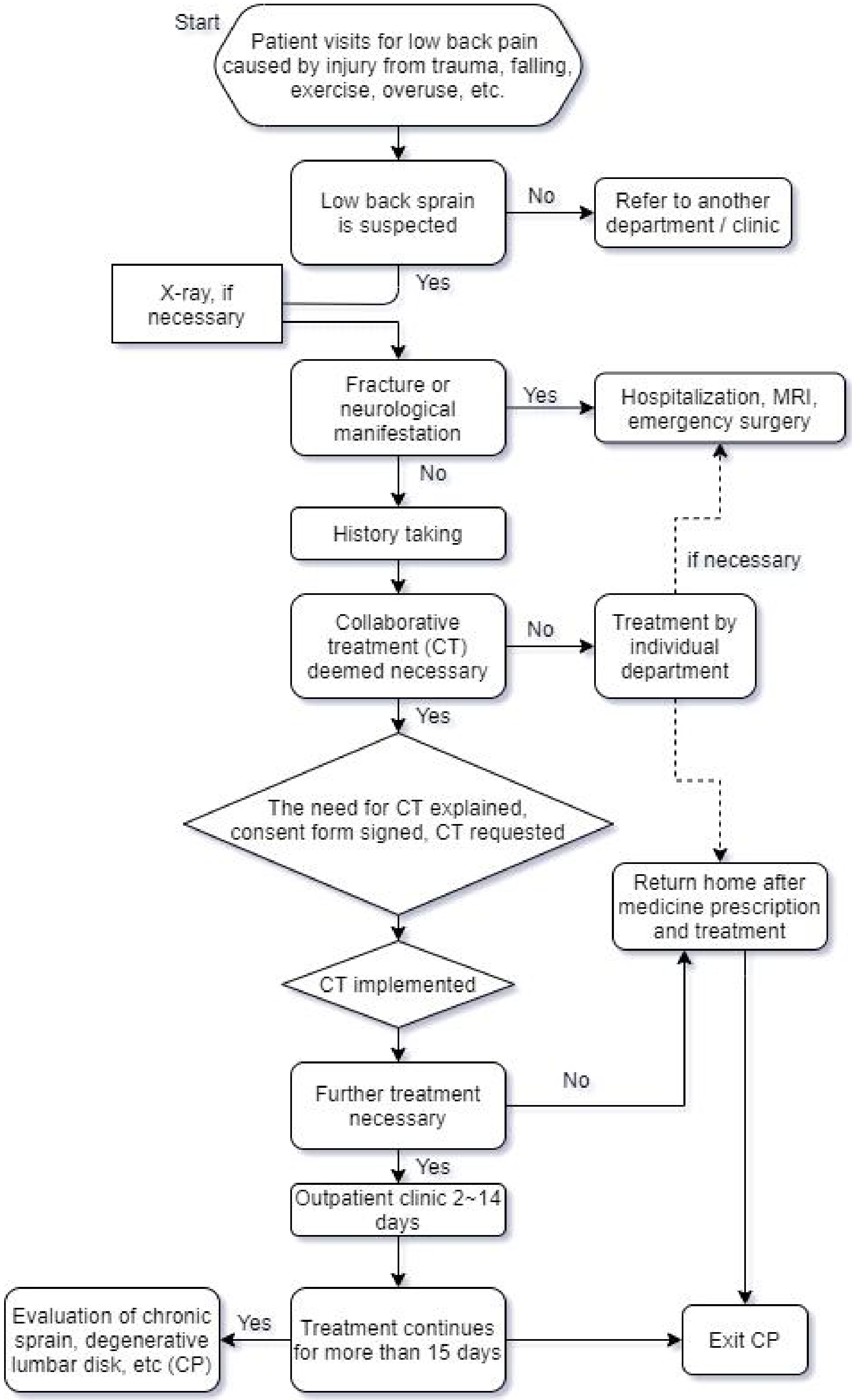

<Treatment Plan>

| | Day 1 | Day 2~14 | After Day 14 |
|---|---|---|---|
| 1. Evaluation & observation | 1. Past medical history, treatment history<br>2. Inspection for damage from injury or trauma<br>3. Physical examination | 1. Progress observation and clinical evaluation | 1. Progress observation and clinical evaluation |
| 2. Examination | 1. X-ray<br>2. Lumbar MRI, if necessary | 1. Lumbar MRI, if necessary | 1. Examination for differential diagnosis |
| 3. CT | 1. CT request / reply<br>2. f/u 2 weeks after the first visit | 1. CT request / reply | 1. CT request / reply |
| 4. Prescription | 1. WM : medication, injection<br>2. KM : herbal medicine, herbal acupuncture | 1. WM : medication, injection<br>2. KM : herbal medicine, herbal acupuncture | 1. WM : medication, injection<br>2. KM : herbal medicine, herbal acupuncture |
| 5. Treatment | 1. WM : physical therapy, kinesitherapy<br>2. KM : acupuncture, KM physiatrics, Chuna | 1. WM : physical therapy, kinesitherapy<br>2. KM : acupuncture, KM physiatrics, Chuna | 1. WM : physical therapy, kinesitherapy<br>2. KM : acupuncture, KM physiatrics, Chuna |
| 6. Education and explanation | 1. Explain treatment and prognosis<br>2. Posture and exercise education | 1. Explain treatment and prognosis<br>2. Posture and exercise education | 1. Explain treatment and prognosis<br>2. Posture and exercise education |

## 3. Clinical pathway for collaborative treatment of lumbar herniated intervertebral disc

- Dong-eui University Korean Medicine Hospital

<Clinical Pathway process>

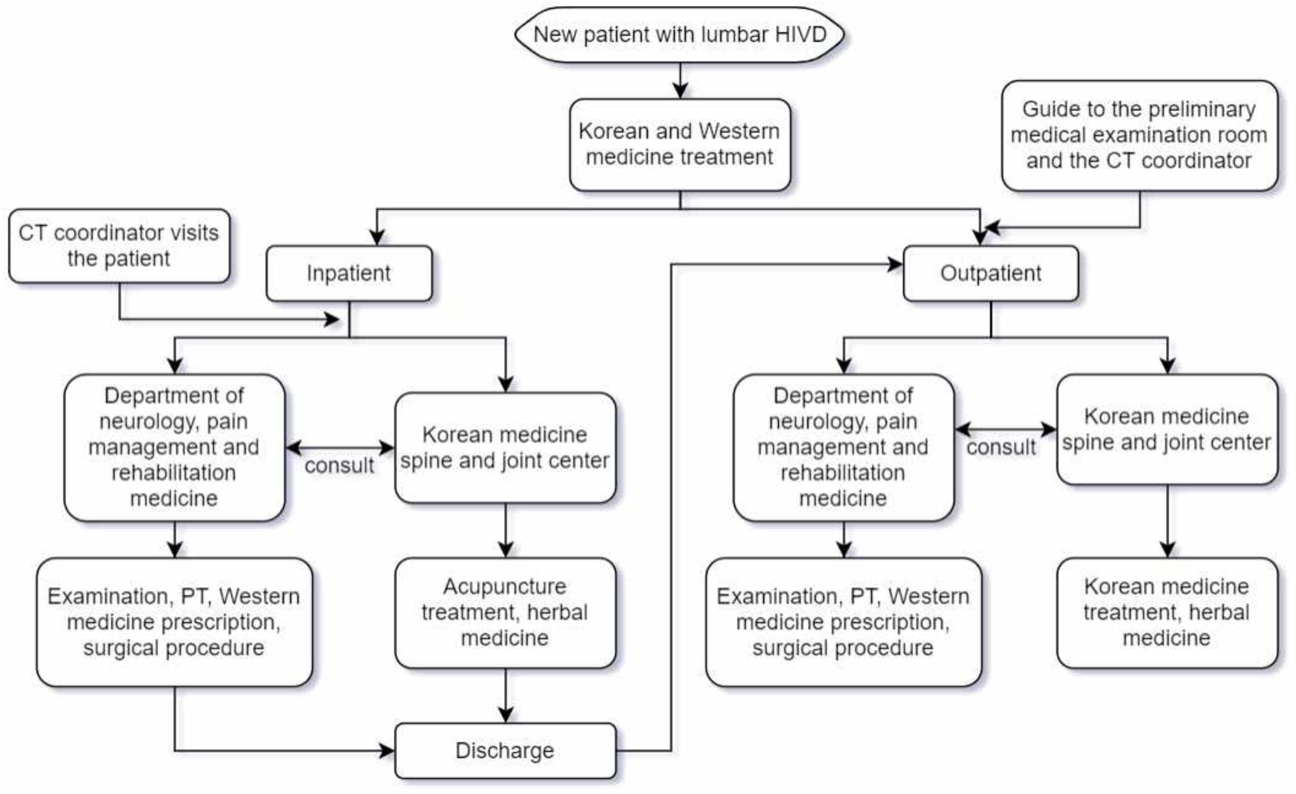

<Treatment Plan>

| | Day 1 | Day 2~14 | Day 15~30 |
|---|---|---|---|
| 1. Evaluation & observation | 1. Past medical history, treatment history<br>2. Physical exmination<br>3. Evaluation of the severity of pain and daily function<br>4. V/S measurement | 1. Evaluation of the severity of pain and daily function<br>2. V/S measurement | 1. Evaluation of the severity of pain and daily function<br>2. V/S measurement |
| 2. Diet | Ordinary diet (no drinking or smoking) | Ordinary diet (no drinking or smoking) | Ordinary diet (no drinking or smoking) |
| 3. Examination | 1. X-ray<br>2 CT or MRI | | |
| 4. Prescription | 1. WM : medication, regional nerve block, etc<br>2. KM : herbal medicine, powdered medicine | 1. WM : medication, regional nerve block<br>2. KM : herbal medicine, powdered medicine | 1. WM : medication, regional nerve block<br>2. KM : herbal medicine, powdered medicine |
| 5. Treatment | 1. WM : physical therapy (electric stimulation), kinesitherapy, manual therapy<br>2. KM : acupuncture, moxibustion, cupping therapy, acupotomy, herbal acupuncture, embedding acupuncture, Chuna, KM physiatrics | 1. WM : physical therapy (electric stimulation), kinesitherapy, manual therapy<br>2. KM : acupuncture, moxibustion, cupping therapy, acupotomy, herbal acupuncture, embedding acupuncture, Chuna, KM physiatrics | 1. WM : physical therapy (electric stimulation), kinesitherapy, manual therapy<br>2. KM : acupuncture, moxibustion, cupping therapy, acupotomy, herbal acupuncture, embedding acupuncture, Chuna, KM physiatrics |
| 6. Education and explanation | 1. Explain treatment and prognosis<br>2. Daily habit training and posture correction<br>3. Exercise education | 1. Explain treatment and prognosis<br>2. Daily habit training and posture correction<br>3. Exercise education | 1. Explain treatment and prognosis<br>2. Daily habit training and posture correction<br>3. Exercise education |

## 4. Clinical pathway for collaborative treatment of lumbar intervertebral disc disorder

- Maru Korean Medicine Hospital

<Clinical Pathway process>

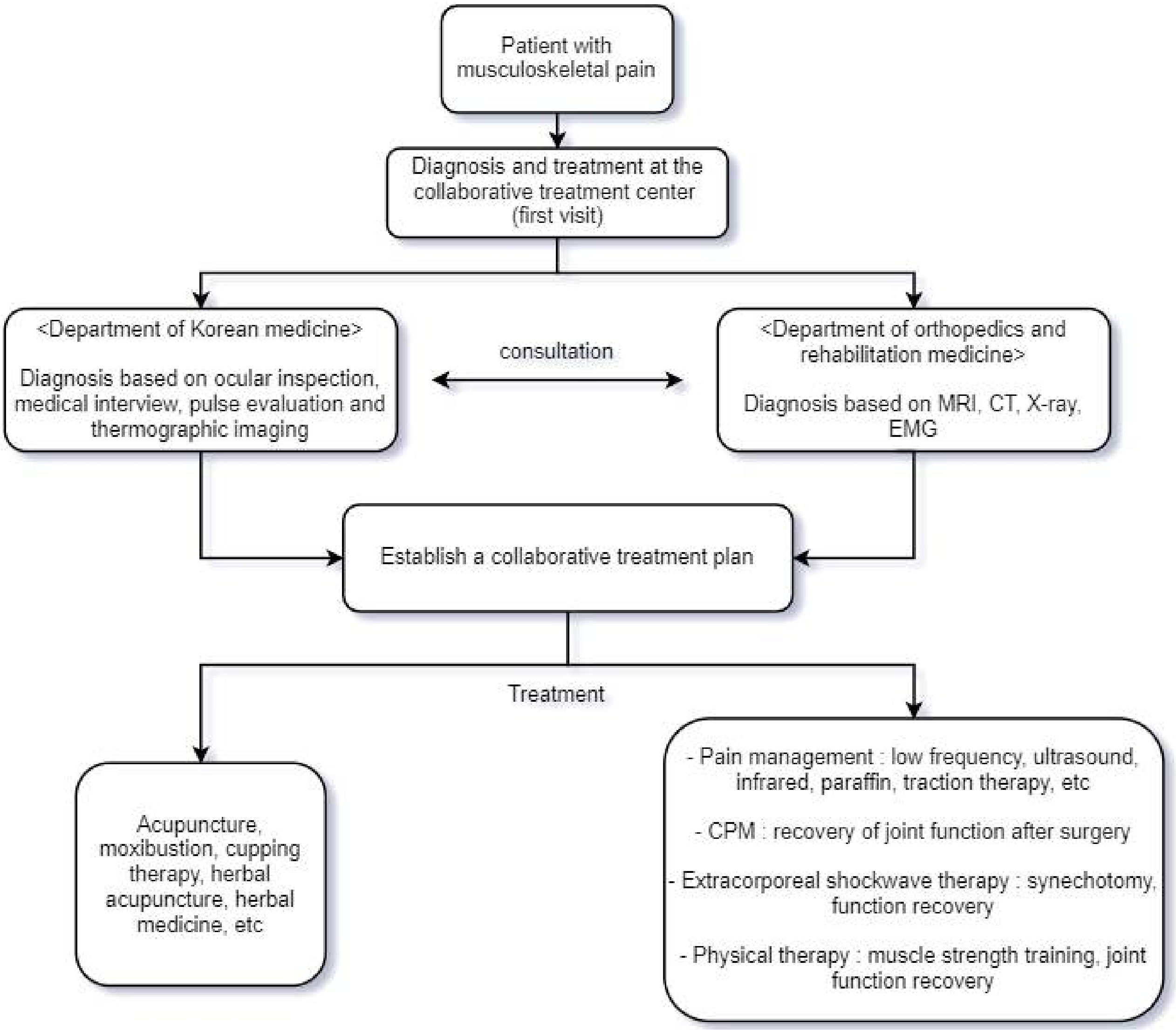

<Sports injry Treatment Plan>

| | Day 1 | Day 2~14 | Day 15~30 |
|---|---|---|---|
| 1. Evaluation & observation | 1. Past medical history, treatment history<br>2. Evaluation of criticality of sports in patient's life<br>3. Acuity or chronicity of the sports injury | 1. Evaluation of recovry from acute damage | 1. Evaluation of possibility of return to sports |
| 2. Diet | Ordinary diet (no drinking or smoking) | Ordinary diet (no drinking or smoking) | Ordinary diet (no drinking or smoking) |
| 3. Examination | 1. X-ray, CT, MRI, EMG, etc<br>2. Diagnosis from ocular inspection, pulse, patient interview, thermographic imaging | | |
| 4. Prescription | 1. WM : steroids, antiphlogistic, analgesic<br>2. KM : herbal medicine | 1. WM : steroids, antiphlogistic, analgesic<br>2. KM : herbal medicine | 1. WM : steroids, antiphlogistic, analgesic<br>2. KM : herbal medicine |
| 5. Treatment | 1. WM : physical therapy (electric stimulation), kinesitherapy<br>2. KM : acupuncture, KM physiatrics | 1. WM : physical therapy (electric stimulation), kinesitherapy<br>2. KM : acupuncture, KM physiatrics | 1. WM : physical therapy (electric stimulation), kinesitherapy<br>2. KM : acupuncture, KM physiatrics |
| 6. Education and explanation | 1. Explain treatment and prognosis<br>2. Education to recover damaged muscles, joints and tendons, and education to prevent recurrence | 1. Explain treatment and prognosis<br>2. Education to recover damaged muscles, joints and tendons, and education to prevent recurrence | 1. Explain treatment and prognosis<br>2. Education to recover damaged muscles, joints and tendons, and education to prevent recurrence |
